# In Vivo MRI-CEST Tumor pH Imaging Detects Resistance to Proton Pump Inhibitors in Human Prostate Cancer Murine Models

**DOI:** 10.3390/cancers14194916

**Published:** 2022-10-07

**Authors:** Pietro Irrera, Lorena Consolino, Miriam Roberto, Martina Capozza, Chetan Dhakan, Antonella Carella, Alessia Corrado, Daisy Villano, Annasofia Anemone, Victor Navarro-Tableros, Martina Bracesco, Walter Dastrù, Silvio Aime, Dario Livio Longo

**Affiliations:** 1 Department of Environmental, Biological and Pharmaceutical Sciences and Technologies, University of Campania “Luigi Vanvitelli”, 81100 Caserta, Italy; 2 Institute of Biostructures and Bioimaging (IBB), National Research Council of Italy (CNR), 10126 Turin, Italy; 3 Department of Nanomedicines and Theranostics, Institute for Experimental Molecular Imaging, RWTH Aachen University, 52074 Aachen, Germany; 4 Department of Molecular Biotechnology and Health Sciences, University of Torino, 10126 Turin, Italy; 5 IRCCS SDN SynLab, 80143 Naples, Italy

**Keywords:** Magnetic Resonance Imaging (MRI), pH, imaging, Chemical Exchange Saturation Transfer (CEST), prostate cancer, Proton Pump Inhibitors (PPIs), iopamidol, treatment, resistance, acidosis, tumor, glycolysis

## Abstract

**Simple Summary:**

Tumor acidosis plays a major role in tumor aggressiveness, invasion and resistance, and it is considered an important target for novel anticancer strategies. In this work, we investigated the therapeutic efficacy of several proton pump inhibitors (Esomeprazole, Lansoprazole, Amiloride and Cariporide) to alter tumor acidity in prostate murine cancer models. The in vitro results showed a moderate toxicity for Esomeprazole that was selected for the successive in vivo studies. However, the in vivo studies highlighted the lack of response to Esomeprazole treatment in both subcutaneous and orthotopic PC3 prostate cancer models. Overall, MRI-based tumor pH imaging is a powerful tool for monitoring the in vivo response to treatment.

**Abstract:**

The tumor microenvironment acidification confers treatment resistance; therefore, the interference with pH regulating systems is considered a new therapeutic strategy. In this study, two human prostate cancer cell lines, PC3 and LNCaP, have been treated in vitro with proton pump inhibitors (PPIs), namely Lansoprazole, Esomeprazole (V-ATPases-inhibitors), Cariporide, and Amiloride (NHE1-inhibitors). The cell viability and pH were assessed at several drug concentrations either at normoxic or hypoxic conditions. Since Esomeprazole showed the highest toxicity towards the PC3 cancer cells compared to LNCaP ones, athymic nude mice bearing subcutaneous or orthotopic PC3 tumors were treated with Esomeprazole (dose: 2.5 mg/kg body weight) for a period of three weeks—and tumor growth was monitored. MRI-CEST tumor pH imaging with Iopamidol was performed upon treatment at 3 h, 1 week (in combination with FDG-PET), and after 2 weeks for evaluating acute, early, and late responses. Although acute tumor pH changes were observed in vivo, long-term studies on both PC3 prostate cancer models did not provide any significant change in tumor acidosis or tumor growth. In conclusion, this work shows that MRI-CEST tumor pH imaging is a valuable tool for assessing the in vivo treatment response to PPIs.

## 1. Introduction

Tumor lesions have common characteristics among different types of cancer, including genetic mutations, altered metabolism, hypoxia, and extracellular acidosis. Furthermore, these characteristics yield conditions that sustain tumor development at the expense of normal tissues that are unable to cope with such extreme conditions [1]. Experimentally, these common traits can be used as useful biomarkers for diagnosis and staging. Moreover, the molecular and cellular mechanisms responsible for the observed behavior can be considered as potential targets for cancer treatment [2,3,4].

Healthy cells maintain a well-balanced metabolism to keep energy consumption and pH homeostasis at physiological levels. However, upon neoplastic transformation, this balance is disrupted, thereby leading to an abnormal pH regulation that favors the establishment of an acidic microenvironment in the tumor tissue [5]. Furthermore, tumor acidosis is responsible for tumor growth, immune resistance, and invasiveness [6,7], thus depicting acidosis as a valuable target for anti-cancer treatments. Studies have shown how tumor alkalinization through systemic administration of bicarbonate can reduce the invasiveness and increase the immune response when coupled with immunotherapy [8,9,10]. Moreover, since the pH balance is maintained thanks to the activity of proton transporter proteins, also called proton pumps—such as carbonic anhydrases (CAs), Na+/H+ exchanger (NHE-1), and vacuolar-ATPase (V-ATPase)—selective targeting of these proteins has been explored as a novel anticancer strategy to alter the acidity of the tumor microenvironment. In particular, proton pump inhibitors (PPIs) are molecules able to inhibit the proton’s exchange toward the extracellular environment and eventually affect the tumor growth or its resistance to other treatments [11,12,13].

Previous pre-clinical investigations with PPIs showed promising results in different tumor models, either when the PPIs were administered alone or together with a chemotherapy drug [14,15,16]. On the other hand, few studies have been conducted so far on prostate cancers with contrasting results. The combined use of Pantoprazole with Docetaxel or with vitamin C led to better therapeutic outcomes compared to the monotherapy alone [17,18]. In general, PPIs treatment yields the expected effects of a more alkaline pH that leads to an enhanced response to immunotherapies [19]. In contrast, a study on several prostate cancer cell lines showed that the treatment with Lansoprazole was actually enhancing the tumor growth both in vitro and in vivo [20].

Imaging techniques, especially magnetic resonance imaging (MRI), are widely used for prostate cancer detection, staging, and guided surgery, and for assessing the inherent heterogeneity [21,22]. Among the variety of biomarkers that can be explored, pH is highly relevant in tumor studies, especially for treatments that induce pH changes [23,24]. Several MR-based methods to measure tumor pH have been proposed, namely 31P-MRS [25,26], 13C Hyperpolarized-MRI [27,28,29] or Chemical Exchange Saturation Transfer (CEST)-MRI technique [30,31]. They differ in their sensitivity, spatial resolution, and pH accuracy [32]. In this context, good progress has been achieved with the use of the pH-responsive contrast agent, Iopamidol, which showed that the extracellular tumor pH can be quantified accurately and with high spatial resolution [33,34,35]. Interestingly, this method has already been translated into patients [36]. Of note, it allowed for the assessment—for the first time in vivo in a murine breast cancer model—of the relationship between a higher glucose consumption rate and increased tumor acidosis [37] and demonstrated a strong association between the acidity of the tumors and their higher invasiveness in several murine breast cancers [38].

In addition, several studies have been carried out recently with this approach to assess the efficacy of novel anticancer therapies affecting tumor metabolism or acidosis [30,39]. Dichloroacetate, a glycolysis inhibitor, has been shown to increase the extracellular pH upon reduced glycolytic flux in breast murine tumors [40], whereas UK-5099, an inhibitor of the mitochondrial pyruvate carrier, provided a decrease in breast murine tumor pH values since more pyruvate is converted to lactate [41]. Furthermore, Metformin caused a decrease in the extracellular tumor pH, thereby reflecting the inhibition of the consumption of cytosolic lactic acid [42]. On the other hand, Syrosingopine, an inhibitor of monocarboxylate transporters (MCT1 and MCT4), did not provide any change in the extracellular tumor pH in the MDA-MB-231 breast model [43]. Similar results were observed upon Dichloroacetate treatment in a triple-negative breast cancer murine model with no changes in tumor acidification [44].

The aim of this work is to evaluate the efficacy of several proton pump inhibitors in two human prostate cancer cell lines (PC3 and LNCaP) by MRI-CEST tumor pH imaging. The cell viability, extracellular pH, and V-ATPase expression (by qRT-PCR and WB) have been measured in cellulo in both normoxic and hypoxic conditions. In addition, in vivo studies in both PC3 subcutaneous and orthotopic murine tumor models have been conducted and tumor growth and extracellular tumor pH have been measured at several time points to assess the response to Esomeprazole treatment.

## 2. Materials and Methods

### 2.1. Cell Culture

Human prostate cancer cell lines, PC3 and LNCaP, were used for the experiments. PC3 cells were grown in D-MEM F12 and LNCaP cells in RPMI 1640, and both media were supplemented with 10% (*v*/*v*) fetal bovine serum (FBS), 100 U/mL penicillin, and 100 mg/mL streptomycin; D-MEM F12, RPMI 1640, FBS, and Trypsin, were purchased from Lonza (Lonza Sales AG, Verviers, Belgium). The penicillin–streptomycin mixture was purchased from Sigma Chemical Co., St. Louis, MO, USA. Cells were incubated in 175 cm^2^ flasks in a humidified 5% CO2 incubator at 37 °C. At confluence, PC3 and LNCaP cells were detached by adding 2 mL of Trypsin–EDTA solution [0.25% (*w*/*v*) Trypsin- 0.53 mM EDTA].

### 2.2. In Cellulo Treatment with Proton Pump Inhibitors

All the inhibitory drugs were purchased from Sigma Chemical Co. (St. Louis, MO, USA). For each inhibitor, a mother stock solution was prepared by dissolving the solid drug in DMSO. For in vitro experiments, the final drug concentrations were obtained by adding 3 µL from the stock solution with different volumes of cell culture medium just before the treatment. For both cell lines, a total of 3 × 10^4^ cells were seeded into 96-well black plates and incubated overnight to allow for adherence in a standard CO_2_ incubator. For each well, a total volume of 100 µL of drug solution was used for treating the cells after the overnight incubation. Treatment times of 24 or 48 h were applied for the cell viability tests, whereas for the pH measurements, there was only a 24 h treatment time. Parallel experiments were performed in hypoxic conditions, thereby incubating the cells in a hypoxic incubator (New Brunswick™ Galaxy^®^ 48 R, Eppendorf S.r.l., Milan, Italy) set to 1% O_2_, 5% CO_2_, and 95% humidity.

### 2.3. Cell Viability Assay

Cell viability tests were conducted with the colorimetric assay of the MTT dye. Specifically, 3-(4,5-dimethylthiazol-2-yl)-2,5-diphenyltetrazolium bromide is converted to its formazan insoluble derivate through NAD(P)H-dependent cellular oxidoreductase enzyme reduction. An MTT solution was prepared by dissolving the yellow powder in PBS and then used after the treatment period of 24 or 48 h. Before adding the MTT solution, each well was washed twice with PBS, then filled with 100 µL of the dye solution and incubated for 4 h in a normoxia or hypoxia incubator. During the incubation, the enzyme reaction produced the formazan crystals (purple colored) that were dissolved with DMSO for the fluorescence reading. Usually, a wavelength between 500 and 600 nm was used to obtain fluorescence measurements that report the amount of produced formazan, and in turn the metabolic activity of the cells.

### 2.4. Extracellular pH Measurement

pH measurements were performed in normoxic conditions with the pH-Xtra Glycolysis Assay (Luxcel Bioscience, Cork, Ireland) kit including Respiration Buffer (RB) and Fluorescent Probe (FP) using a microplate reader (BioTek Instruments, Inc., Winooski, VT, USA). Following the manufacturer’s protocol, 96-well plates were removed after the treatment period from the standard incubator (5% CO_2_, 37 °C) and maintained in a CO_2_-free incubator at 37 °C for two hours to allow the gas to purge. In the next step, the growth medium was removed, and each well was washed twice with RB and then filled with the FP-RB solution (1:10, total volume per well 100 µL). Prior to its use, the RB tablet was resuspended in 50 mL of bi-distilled water titrated to pH 7.4 with NaOH 1M and then filtered with 0.2 µm of filter, whereas the FP was resuspended in 1 mL of bi-distilled water. The fluorescence intensity was measured kinetically for two hours, ending with a dual-read time-resolved fluorescence mode and keeping the reader at 37 °C. This dual-read mode takes into account the fluorescence decay by measuring the fluorescence intensity with two different delays for data collection, thereby allowing for the calculation of the lifetime (µs) for each well with the formula [D_2_ − D_1_/ln(R_1_/R_2_)]—where D_1-2_: delay 1 = 100 µs, delay 2 = 300 µs; R_1-2_: read 1, read 2. Finally, it was possible to derive the pH values using the calculated lifetime values from the measured calibration curve by using this formula: pH = a (lifetime) + c. The calibration curve was obtained by titrating the Respiration Buffer at different pH values and plotting the lifetime values obtained with the double-read approach for each experimental pH value.

### 2.5. qRT-PCR

TRIzol^®^ reagent (15596018, Invitrogen, Waltham, MA, USA) was used to extract the total RNA from the PC3 cell line according to the manufacturer’s instructions. cDNA was synthesized from total RNA (500 ng) using the SuperScript III Reverse Transcriptase Kit (Invitrogen, 18080044) by following the manufacturer’s recommendations. The quantitative RT-PCR reaction was performed using Sybr Green 2× PCR Master Mix (4309155, Applied Biosystems, Waltham, MA, USA) and the oligonucleotides listed in Table S1. qRT-PCR was carried out on a 7900HT Fast Real-Time PCR System (Applied Biosystems). GAPDH was used to standardize the data, as the house-keeping gene, by following the ΔΔCt method.

### 2.6. Western Blot

Protein extraction was performed using RIPA Lysis buffer (Merk Millipore #20-188, Burlington, MA, USA) supplemented with Protease Inhibitor Cocktail (Sigma #P2714). The protein concentrations were measured with a Thermo Fisher Pierce BCA Protein Assay Kit (Thermo-Fisher, #23225, Waltham, MA, USA) and 50 g of total protein was separated by Bio-Rad Mini-PROTEAN^®^ TGX ^TM^ Gel (Bio-rad #456-9034). Proteins were transferred to a 45-μm-pore polyvinylidene difluoride (PVDF) membrane (Immobilon PSQ, Millipore) and the membrane was blocked in TBS-T (Tris-buffered saline with 0.1% Tween-20) with 5% milk. Primary antibodies for ATP6V1A (1:1000; Abcam #137574, Cambridge, UK) and β-actin (1:3000; Sigma-Aldrich #A1978) were detected by anti-rabbit IgG (1:5000; Sigma # A6154) and anti-mouse IgG (1:5000; Sigma #A4416). The signals were detected with the Pierce TM ECL Western Blotting Substrate kit (Thermo-Fisher #32106) and the subsequent bands were quantified with the ImageJ software (version 1.53c, https://imagej.nih.gov/ij/index.html).

### 2.7. Cells Conditioning with pH-Adjusted Culture Medium

PC3 cells were conditioned to pH 6.8 using a specifically modified medium to maintain such a pH value. This condition was used to mimic the acidic tumor extracellular environment and also to increase the efficacy of drug treatment since Esomeprazole is activated at low pH [45]. Powder medium was used and 25 mM of HEPES and PIPES were added during its dissolution in sterile water. Once all the powdered compounds dissolved in water, the pH was adjusted to the desired value with HCl 3M. Prior to its use, conditioned medium was filtered with a 0.22 µm filter and then completed, as reported previously for the standard medium. Once standard PC3 reached a 70–80% confluence, the standard medium was removed and substituted with the conditioning one. PC3 cells were considered “conditioned” once they reach a similar replication rate compared to the standard ones.

### 2.8. Experimental In Vivo Settings

All animal procedures and husbandry were performed in accordance with our University Ethical Committee and European guidelines under directive 2010/63. Athymic nude mice were purchased from Envigo Srl (San Pietro al Natisone, Italy) and housed in a temperature-controlled room with a 12-h light/12-h dark schedule and fed with autoclaved chow and water ad libitum. After 8 to 10 weeks, male mice (N = 24) were subcutaneously inoculated in both flanks with 1 × 10^6^ PC3 cells. During tumor development, the volumes were recorded with caliper measurements by taking the two dimensions of height and length. The formula $V=\frac{H\;\times \;L^{2}}{2}\;$ was used to calculate the tumor volume values. For the orthotopic model, 1 × 10^6^ PC3 cells were injected into the prostate frontal lobe (N = 8 mice). A small cutting was performed in the low abdomen to expose the injection site, which was sutured after the inoculation. ITK-Snap software (version 3.6, itksnap.org) was used to measure the tumor volumes from the T_2_-weighted axial images, where an ROI was placed on the tumor region for every slice that was found. Imaging sessions started when tumors reached 4–5 mm in diameter. During the MRI experimental setup, mice were maintained under systemic anesthesia that was provided by the mixture of tiletamine/zolazepam 20 mg/kg (Zoletil 100; Virbac, Milan, Italy) and xylazine 5 mg/kg (Rompum; Bayer, Milan, Italy) and injected intramuscularly. Esomeprazole powder was resuspended in saline solution (NaCl 0.9%) with a final concentration of 1 mg/mL and administered every other day by gavage at a dose of 2.5 mg/kg body weight. The treatments started once the tumors reached dimensions of 50–80 mm^3^ and an average volume of 100 µL was given for each administration.

### 2.9. MRI In Vivo Experiments

All images were acquired on a Bruker 7T Advance Neo MRI scanner (Bruker Biospin, Ettlingen, Germany). B_1_ = 3 µT, TS_1_ = 3 s, TS_2_ = 1 s were selected for the saturation module. The image matrix was 128 × 218 for a field of view of 30 mm × 30 mm, with a slice thickness of 1.5 mm and 8 slices [35]. Other acquisition parameters were PFF = 1.6, TR = 11,256 ms, and TE = 3.87 ms. For 3D in vivo CEST experiments, mice were anesthetized as previously reported and a tail vein catheter for Iopamidol injection was placed. The respiratory rate was monitored using an air-pillow (SA Instruments, Stony Brook, NY, USA). After acquisition of scout images and of a T_2_-weighted anatomical reference image, the Z-spectra before and after Iopamidol injection (dose: 4 g Iodine/kg b.w. for tumor pH imaging) were acquired and analyzed to calculate the 3D pH maps. For each CEST acquisition, 46 frequency offsets were acquired by varying the center frequency of the CEST RF pulse from −10 to 10 ppm. CEST-pH acquisitions started after one week of treatment with Esomeprazole and were repeated at two weeks after treatment for the subcutaneous model.

### 2.10. CEST Imaging Analysis

All CEST images were analysed using a home-made script implemented in MATLAB (The Mathworks, Inc., Natick, MA, USA). The Z-spectra were interpolated, on a voxel-by-voxel basis by smoothing splines, were B0-shift corrected, and the saturation transfer efficiency (ST%) was measured by punctual analysis. Difference contrast maps (ΔST%) were calculated by subtracting the ST contrast after iodinated contrast media injection from the ST contrast before the injection on a per voxel basis in order to reduce the confounding effect of the endogenous contributions. A threshold value of 1% was set based on the ΔST variations between multiple pre-contrast ST maps to discriminate between enhancing and non-enhancing pixels. pHe values were estimated in vivo after the Iopamidol injection, thereby applying the ratiometric procedure. The pHe maps were superimposed onto the anatomical reference image.

### 2.11. PET Imaging

PET static acquisitions were performed 45 min after the intravenous injection of 18F-FDG (dose of 15 ± 3 MBq). Mice (N = 6) were anesthetized with isoflurane vaporized with O2. The isoflurane concentration was set at 3.0% for induction and at 1.0–2.0% for maintenance. Mice were imaged using the trimodality PET/SPECT/CT Triumph scanner (Trifoil imaging, Chatsworth, CA, USA). Mice were kept fasted overnight before intravenous 18F-FDG injection. PET data were reconstructed using 2D-Maximum Likelihood Expectation Maximization algorithms with 10 iterations and were corrected for tracer decay and for photon attenuation. An analysis of the PET images were performed using PMOD software (version 4.1, http://www.pmod.com). A volume-of-interest approach was used to determine the amount of radiotracer uptake and to determine the regional values for assessing the maximal percentage of the injected dose per cubic centimeter (%ID/cm^3^).

### 2.12. Histological Staining

Formalin-fixed, paraffin-embedded tumor tissues were cut into slices (5 µm thickness) and stained with standard hematoxylin and eosin (H&E). H&E tumor sections were visualized using an Olympus BX41 optical microscope. The tumor necrosis score was based on the histological evaluation of all tumor tissues and calculated as the percentage of necrosis: 0, no necrosis; 1, <25% necrosis; 2, 25–50% necrosis; 3, 50–75% necrosis; 4, >75% necrosis [46].

### 2.13. Statistical Analysis

Data are expressed as mean values with standard deviations (SD) in all graphs. The statistical significance of the in vitro results was determined by ANOVA analysis coupled with Bonferroni post-hoc correction among the different conditions explored. For the in vivo results, an unpaired Student’s *t*-test was applied. The statistical analysis was performed with Graphpad Prism version 9.1 (La Jolla, CA, USA).

## 3. Results

### 3.1. In Vitro Results: Cell Viability

**Figure 1:**
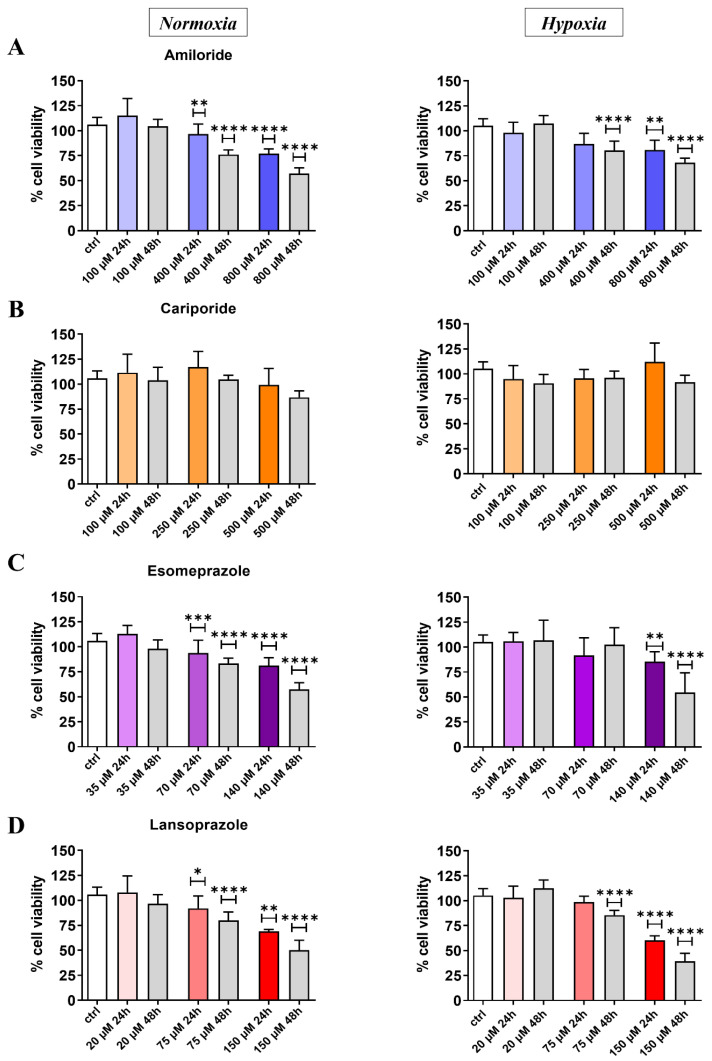
Cell viability results for PC3 cell line in normoxic (left column) and hypoxic (right column) conditions after 24 or 48 h upon treatment at different concentrations with Amiloride (**A**), Cariporide (**B**), Esomeprazole (**C**), and Lansoprazole (**D**), respectively. All drug concentrations are expressed in µM units with the color intensity matching the increasing concentration for the 24 h treatment experiment. ANOVA statistical analysis with Bonferroni post-hoc correction was applied against the control group at the two different time points * *p* value < 0.05; ** *p* value < 0.01; *** *p* value < 0.001; **** *p* value < 0.0001).

The proton pump inhibitors’ effects on the prostate cancer cell lines were first assessed in vitro through the MTT assay. All the screened drugs, except for Cariporide, induced a decrease in cell viability with a more marked effect at 48 h in a dose-dependent trend (Figure 1). Amiloride treatment induced a dose-dependent response in PC3 both at 24 and 48 h in normoxic conditions, with a maximum of 45% of cell deaths observed at the highest drug concentration (800 µM) after 48 h of treatment (Figure 1A). A limited toxic effect was observed in the hypoxic conditions with 30% cell death. Esomeprazole showed a moderate cell death (20–25%) when PC3 cells were tested after 24 h of treatment in normoxic and hypoxic conditions, even at high concentrations. However, 48 h exposure to Esomeprazole revealed a marked response in normoxic and hypoxic conditions, reaching a cell death of 50% with the highest dose (Figure 1C). Its homologue, Lansoprazole, reported similar or even slightly higher toxicity in PC3 cells compared to Esomeprazole at 24 h and 48 h in both normoxic and hypoxic conditions (Figure 1D).

Supplementary Figure S1 shows the MTT results for the LNCaP human prostate cancer cell line. In analogy to the results obtained for the PC3 cell line, Amiloride showed good toxicity effects both in normoxic and even higher in hypoxic conditions (Figure S1A). A similar dose-dependent decrease in cell viability was observed at 48 h after Amiloride treatment in both the tested conditions (Figure S1A). Cariporide was not toxic at all the investigated concentrations (Figure S1B), whereas Esomeprazole induced cell death only at the highest concentration (140 µM), thereby showing a significant decrease in cell viability in both normoxia and hypoxia (normoxia: 83% viability at 24 h, 60% at 48 h; hypoxia: 64% viability at 48 h, Figure S1C). Lansoprazole induced a stronger cell death at the highest concentration (150 µM), thereby decreasing the cell viability below 50% in both normoxic and hypoxic conditions at 24 and 48 h after drug exposure (Figure S1D). Comparing these findings with those for PC3, LNCaP cells showed toxicity mostly at the highest concentrations but without a dose-dependent decrease in cell viability.

### 3.2. In Vitro Results: Extracellular pH

**Figure 2:**
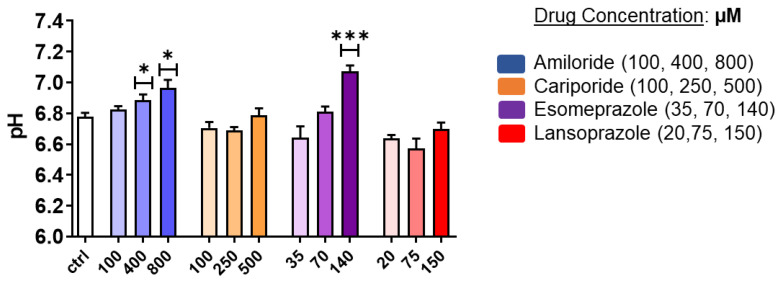
Extracellular pH measurements for PC3 prostate cancer cells after 24 h treatment with Amiloride, Cariporide, Esomeprazole, and Lansoprazole at different concentrations in normoxic conditions. ANOVA statistical analysis with Bonferroni post-hoc correction was assessed (* *p* value < 0.05; *** *p* value < 0.001).

A mild acidic extracellular pH value (ca. 6.8) was measured for PC3 control cells, thereby confirming their tendency to create an acidic environment. pH measurements on treated cells showed a significant extracellular alkalinization for PC3 after 24 h treatment with Esomeprazole and Amiloride (Figure 2). In analogy with MTT experiments, Cariporide showed no effect on pH regulation. Notably, Lansoprazole treatment also induced no changes in extracellular pH. Amiloride was found to alter the extracellular pH in PC3 cells in a dose-dependent manner, thereby providing a statistical difference compared to the control with a pH of 6.89 and 6.97 at 400 and 800 µM, respectively. Together with Amiloride, Esomeprazole treatment provided a strong extracellular basification at the 140 µM concentration, thereby reaching an average pH value of 7.1.

As shown in Supplementary Figure S2, the LNCaP cell line reported a more acidic pH (ca. 6.5 for the control) compared to PC3 cells, but only Esomeprazole induced a significant alkalinization of the extracellular environment towards a pH value of 6.95 at the highest concentration.

### 3.3. In Vitro Results: V-ATPase Expression

**Figure 3:**
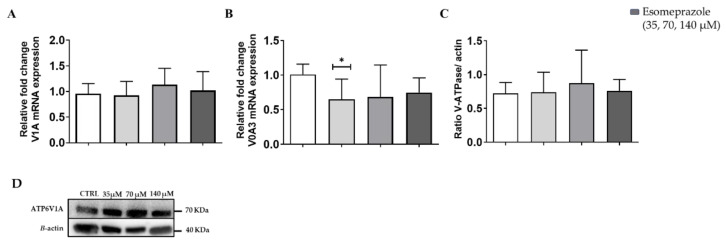
V-ATPase mRNA expression for the subunits V1A (**A**) and V0A3 (**B**); quantification of protein level (**C**) and Western Blot analysis (**D**) of PC3 cells after 24 h Esomeprazole treatment. White columns represent the control; grey-colored columns represent the three different concentrations of esomeprazole. ANOVA statistical analysis with Bonferroni post-hoc correction was applied (* *p* value < 0.05).

Since the in vitro experiments showed that Esomeprazole was the most effective drug in inducing cell toxicity and changes of the extracellular pH in the PC3 cancer cell line, we focused our attention on this drug in the following studies. Further in vitro characterization proceeded with the quantification of the V-ATPase expression on the PC3 prostate cancer cells upon 24 h Esomeprazole treatment. The V1A mRNA expression reported no changes upon Esomeprazole treatment (Figure 3A). In contrast, the transmembrane subunit, V0A3, resulted in decreased expression for all the drug concentrations in cells grown in normal medium (Figure 3B). On the other hand, Western blots against the V1A subunit reported no significant variations in protein levels at every drug concentration compared to control (Figure 3C and Supplementary Figure S3).

### 3.4. In Vivo Results: Acute Esomeprazole Effect in the PC3 Prostate Subcutaneous Model

**Figure 4:**
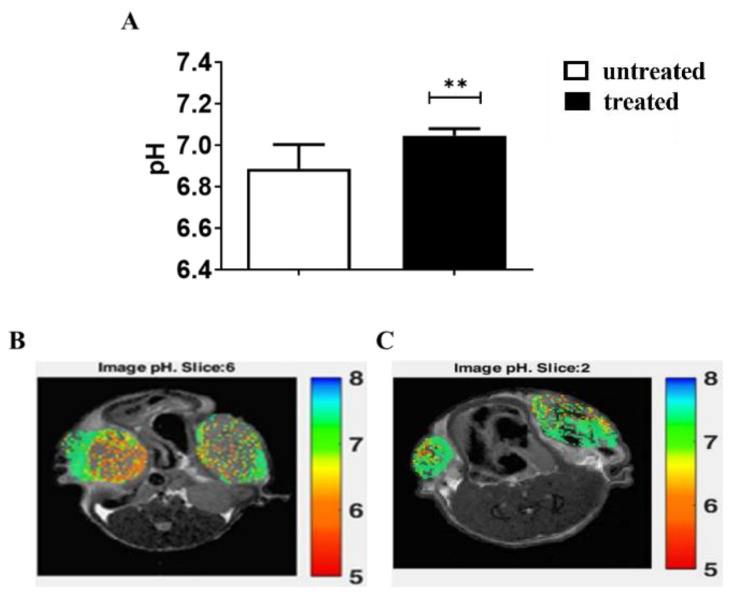
MRI-CEST extracellular tumor pH values for PC3 subcutaneous tumor murine model after 3 h of Esomeprazole administration (dose: 2.5 mg/kg b.w.) by gavage in treated (N = 4) and untreated (N = 5) groups (**A**). The statistical differences resulted from unpaired Student’s *t*-test (** *p* value < 0.01). pH maps showing the pH distribution across tumor regions in a representative untreated mouse (**B**), average tumor pH = 6.82) and in a treated mouse (**C**), average tumor pH = 7.0).

Due to the higher efficacy of Esomeprazole in inducing cell toxicity and changes in the extracellular pH, the in vivo study was carried out in murine models of PC3 prostate tumors. First, we evaluated the acute effect of Esomeprazole on the extracellular tumor pH in the PC3 subcutaneous prostate murine cancer model in vivo. The treatment was done once the tumor reached dimensions of about 80 mm^3^. After three hours from Esomeprazole administration by gavage, mice underwent MRI-CEST pH imaging acquisitions. The pH readouts revealed a significant tendency towards neutralization of the acidic pH (6.86 vs. 7.05, untreated vs. treated, *p* < 0.01) in PC3 tumors (Figure 4A). The representative tumor MRI pHe images for untreated (Figure 4B) and treated mouse (Figure 4C) are shown.

### 3.5. In Vivo Results: Long-Term Esomeprazole Treatment in the Prostate PC3 Subcutaneous Model

**Figure 5:**
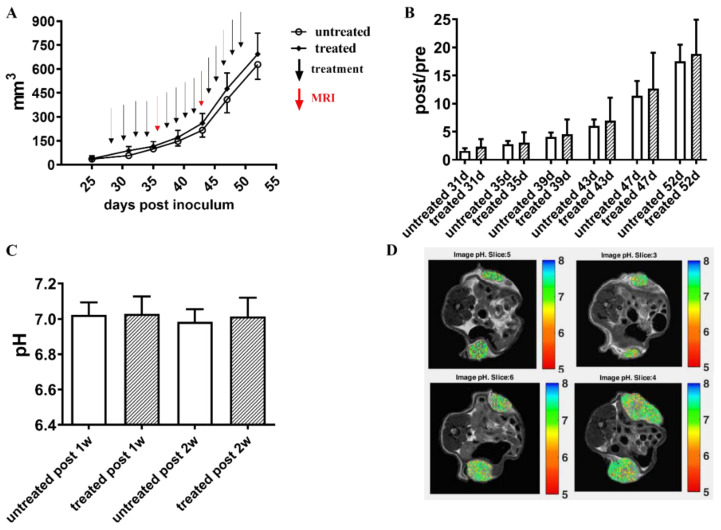
Long-term Esomeprazole treatment in the PC3 subcutaneous tumor model. Tumor growing curves obtained from caliper measurements (**A**). The black arrows indicate the day of intraperitoneal Esomeprazole administration (dose: 2.5 mg/kg b.w., every other day by gavage), whereas red arrows show MRI-CEST pH-imaging acquisitions. Column bar graph showing normalized tumor growth volumes, obtained by dividing the post-treatment volume measurements by the volumes measured on the day of mice randomization into the two groups (**B**). Extracellular tumor pH values between untreated (N = 8) and treated (N = 8) groups (white and pattern-filled, respectively, (**C**)). Representative pH images of untreated and Esomeprazole-treated mice at one week (first row) and two weeks (second row) post-Esomeprazole treatment (**D**).

Since Esomeprazole induced changes in tumor acidosis a few hours after administration, we deemed it of interest to investigate the long-term response of treatment in the same PC3 murine tumor model. Subcutaneously PC3-injected mice developed a palpable tumor mass at the fourth week post-inoculum and were randomly divided into untreated and treated groups. Esomeprazole was given for three weeks, and the MRI-CEST tumor pH images were acquired at 1 week and after 2 weeks of treatment to evaluate the pH response. Figure 5A shows that the tumor growing curve for the untreated and treated PC3 groups are almost superimposed. Furthermore, after the second week of treatment (43 days post-inoculum), the tumor masses increased their growing rate (Figure 5B), thereby doubling their dimensions and reaching the endpoint at an average volume of 650 mm^3^ and 700 mm^3^ for the untreated and treated group, respectively. MRI-CEST pH imaging revealed a mild acidic pH for the untreated group of the PC3 tumor model, and any pH change was detected in the treated group compared to the untreated one over the entire period (Figure 5C). The representative tumor pHe images for untreated and treated mouse after one week of treatment (upper row) and two weeks of treatment (lower row) are shown in Figure 5D.

**Figure 6:**
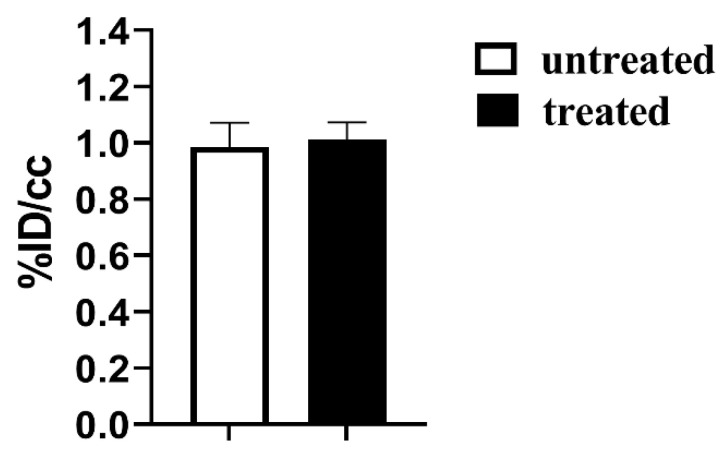
FDG-PET glucose %ID/cc uptake values for the subcutaneous PC3 tumor murine model after one week of treatment with Esomeprazole (dose: 2.5 mg/kg b.w., every other day by gavage) for untreated (N = 3, white bar) and treated (N = 3, black bar) mice.

In addition, FDG-PET images have been acquired in a separate cohort of mice (N = 6, 3 untreated and 3 treated) to further investigate the effect of Esomeprazole in tumor metabolism after one week of treatment. Esomeprazole-treated PC3 mice did not report any change in glucose uptake after one week of treatment compared to the untreated group (Figure 6). Therefore, FDG-PET results were consistent with the observed MRI findings that reported no changes in tumor metabolism and acidosis between the untreated and treated groups.

**Figure 7:**
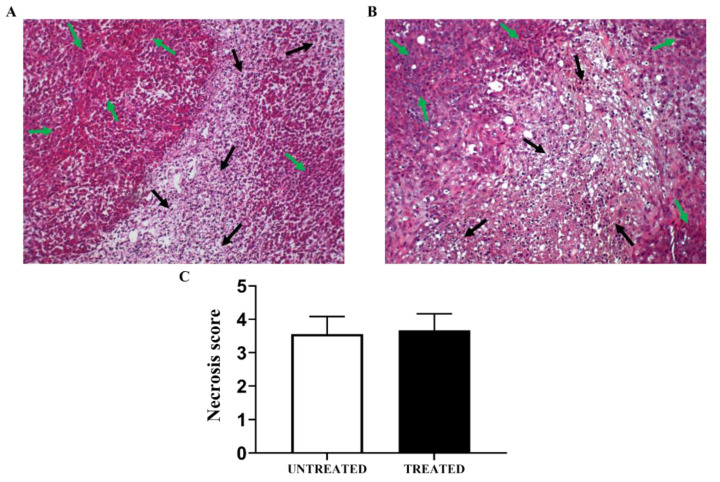
Representative hematoxylin and eosin tumor sections for the PC3 subcutaneous tumor model showing regions of viable tumor (green arrows) and of highly-diffused necrosis (black arrows) in one week of treatment (**A**) and untreated (**B**) PC3 tumors. Bar graph of calculated tumor necrosis score in untreated and treated tumors (**C**).

H&E tumor sections collected from the subcutaneous model after one week of Esomeprazole treatment showed extensive necrotic clusters along the whole tumor tissue. However, necrotic areas were highly diffused across the tumor, with no statistical differences between the untreated and treated PC3 tumors (Figure 7A,B). The tumor necrosis scores showed high but comparable values in both treated and untreated PC3 tumors (Figure 7C).

### 3.6. In Vivo Results: Long-Term Esomeprazole Treatment in the Orthotopic PC3 Prostate Model

**Figure 8:**
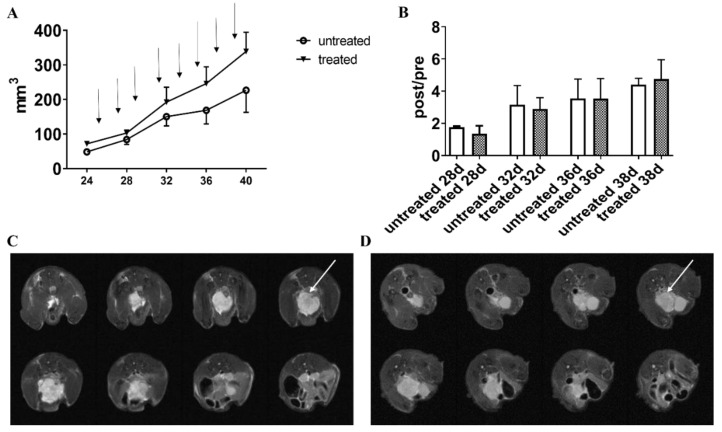
PC3 tumor volumes for the PC3 orthotopic model measured from MRI T_2_-weighted anatomical images. The black arrows indicate the day of Esomeprazole administration (dose: 2.5 mg/kg b.w. by gavage, every other day) (**A**). Normalized tumor volumes (calculated by dividing the post-treatment volume measurements by the volume measured on the day of mice randomization into the two groups, (**B**)). Representative T_2_-weighted anatomical images of orthotopic PC3 prostate tumors (8 slices) of untreated (**C**) and treated mice (**D**) at one week post-Esomeprazole treatment. The white arrows point the tumor mass.

Since orthotopic models, compared to subcutaneous ones, are known to better simulate clinical prostate cancer, particularly regarding the tumor microenvironment, orthotopic PC3 tumor bearing mice were also investigated to assess the treatment response to Esomeprazole. PC3 mice developed an appreciable tumor mass after the third week from cell inoculum. Once tumors reached a volume of around 50 to 80 mm^3^, Esomeprazole was given every other day by gavage at a dose of 2.5 mg/kg b.w. and MRI sessions were performed every 4 days to monitor the tumor volume growth. After two weeks of treatment, orthotopic PC3 treated mice showed larger tumor volumes (Figure 8A). As reported in Figure 8B, the normalized tumor volumes were similar in the untreated and Esomeprazole-treated mice. Figure 8C shows the representative anatomical images of the orthotopic prostate tumors one week after treatment for the two groups.

### 3.7. In Vitro Results: PC3 Conditioned at Acidic pH

**Figure 9:**
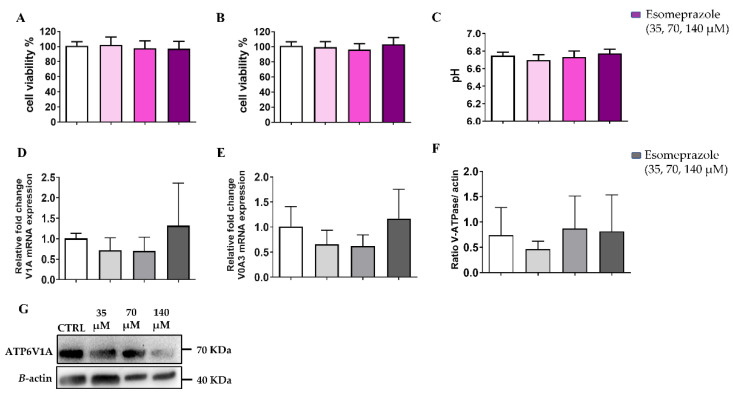
Cell viability assays in normoxia (**A**) and in hypoxia (**B**) and pH measurements (**C**) assays for PC3 acidic-conditioned (pH = 6.8, in normoxia) cells treated with Esomeprazole. V-ATPase mRNA expression for the subunits V1A (**D**) and V0A3 (**E**). Quantification of protein level (**F**) and Western Blot analysis (**G**) of PC3 conditioned at acidic pH after 24 h Esomeprazole treatment. White columns represent the control; grey-or-violet-colored columns represent the three different concentrations of esomeprazole. ANOVA statistical analysis with Bonferroni post-hoc correction was applied.

To get more insight into the lack of response upon Esomeprazole treatment to both tumor growth and acidosis in both subcutaneous and orthotopic PC3 murine cancer models, we deemed it of interest to investigate the behavior of PC3 cells that are chronically conditioned to an acidic environment (pH = 6.8). These conditioned PC3 cells appeared to be a good model for mimicking the highly acidic microenvironment measured in in vivo tumors. PC3 cells were conditioned to pH 6.8 by replacing the standard culture medium with a buffered conditioning medium. After several medium replacements and cell passages, PC3 started to better tolerate the permanent acidic pH and were used for the experiments once they reached a similar replication rate to standard PC3. In the acidic-conditioned PC3 cells, we did not observe differences in the cell viability for the three investigated concentrations of Esomeprazole both in normoxia and hypoxia (Figure 9A and Figure 9B, respectively). This loss of effect was also found in the pH measurements that reported no changes in the extracellular acidification after the treatment (Figure 9C).

The expression of the two subunits, V1A and V0A3, did not change upon Esomeprazole treatment for the acidic-conditioned PC3 cells (Figure 9E and Figure 9F, respectively). Similarly, the V-ATPase protein expression was also not affected by Esomeprazole at increasing concentrations (Figure 9F and Supplementary Figure S3).

## 4. Discussion

In this study, the efficacy of proton pump inhibitors was tested in vitro and in vivo on two human prostate cancer cell lines, namely PC3 and LNCaP. Na^+^/H^+^ Exchanger-1 (also known as sodium-hydrogen antiporter 1) and V-ATPase (vacuolar-ATPase) were targeted with Amiloride, Cariporide, Esomeprazole, and Lansoprazole, respectively, at three concentrations. The treatment response was assessed via in vitro tests of cell viability and pH measurements. V-ATPase activity involves different cellular mechanisms in prostate cancer and its inhibition implies impairment of the apoptosis process and cell cycle, and variation in prostate-specific antigen (PSA) levels and expression [47]. Consequently, drugs targeting V-ATPases can alter the aggressiveness and the invasion ability of prostate cancer cells. NHE-1 is usually highly expressed in prostate cancers and correlates with the expression of transcription factors, such as Zeb1, which promote the mesenchymal transition and, consequently, the invasion and metastatic potential of the tumor lesions [48]. Although more studies are needed to fully depict the roles of these transporters, they are already considered a valuable target to test new approaches for cancer therapies [49].

MTT experiments provided an overview of the inhibitory effects that reported a cell viability decrease for all the used drugs, except for Cariporide, which was found to be ineffective at all of the concentrations and conditions investigated. Furthermore, the inhibitory effects for all the drugs (except Amiloride) were more pronounced after 48 h of treatment compared to 24 h, whereas only Amiloride resulted in less effective treatment, thereby decreasing cell viability. Overall, the human prostate PC3 cancer cell line showed a mild tolerance to drug toxicity when experiments were carried out in hypoxic compared to normoxic conditions. On the other hand, the reduced toxic effects were observed for the LNCaP prostate cancer cells with all the tested inhibitors, particularly in normoxic conditions, wherein only the highest doses produced significant decreases in cell viability.

Extracellular pH measurements in PC3 cells revealed a significant pH modification upon Amiloride treatment in the PC3 cells that led the extracellular environment towards a neutral pH value (6.8 vs. 7.0 for control and treated cells, respectively). An even more pronounced basification occurred after Esomeprazole treatment (6.8 vs. 7.1, control and treated cells, respectively). For the LNCaP cells, although being more prompt to acidify the extracellular environment in comparison to the PC3 cells, PPI treatments revealed a significant alkalinization of the extracellular space only at the highest dose of Esomeprazole (6.5 vs. 6.95, control vs. treated), with no pH modification for the other investigated inhibitors. On the basis of these findings, Esomeprazole was selected for the subsequent in vivo studies. First, the in vivo experiments were carried out with a PC3 subcutaneous model to assess the acute effect of Esomeprazole on tumor acidosis. After three hours after administration, Iopamidol-based MRI-CEST pH imaging showed a significant trend to more alkaline pH values in Esomeprazole-treated mice. On the other hand, the long-term administration with Esomeprazole was unable to reduce the tumor growth in both the subcutaneous and the orthotopic models after two and three weeks of treatment. Furthermore, no extracellular tumor pH changes were observed after one week and two weeks of treatments in all the imaged mice. In addition, FDG-PET imaging confirmed no changes in the uptake of glucose between treated and untreated groups after one week of Esomeprazole treatment. Therefore, in contrast to the in vitro studies and to the observed acute effect on extracellular tumor pH, one may draw the conclusion that PC3 humane prostate cancer cells are resistant to Esomeprazole in vivo. This lack of therapeutic effect seems to not be dependent on a low concentration achieved inside the tumor, since the acute effect study demonstrated that enough accumulation was followed by a therapeutic response. Other explanations could rely on the overexpression of proton transporters other than V-ATPases, or compensatory mechanisms that reduce the stress induced by V-ATPase [50].

When the PC3 cancer cells were conditioned to pH 6.8 using a buffered culture medium to mimic the chronic stress conditions usually found in the acidic tumor microenvironment, any effects on cell viability and on the extracellular pH value were observed after 24 h of Esomeprazole treatment. WB and PCR analyses performed on non-conditioned and acidic-conditioned PC3 cells reported no differences in the amount of mRNA expression and protein levels upon Esomeprazole treatment. No changes in V-APTase expression were previously observed when treating human melanoma cell lines with PPIs [16], whereas both Esomeprazole and Pantoprazole reduced the V-ATPase expression in human gastric adenocarcinoma cell lines [51]. The absence of variation in the V-ATPase expression could be associated with a shift of subcellular localization.

Numerous studies have been conducted with proton pump inhibitors in several types of tumors and reported promising results for tumor inhibition, wherein these inhibitors have been used alone or in combination with chemotherapeutic drugs—even at the clinical level. All these studies carried out in lymphoma, breast, or colorectal cancers showed that the PPIs were effective in reducing tumor growth in in vivo models as well [17,52,53,54,55,56,57,58]. In a recent study, Gesmundo and co-workers observed a moderate resistance in PC3 and DU145 prostate cancer cell lines upon treatment with Omeprazole, Lansoprazole, and Esomeprazole (at lower doses than those used in our study, with a maximum concentration of 5 µM) [59]. We observed similar findings only at the lowest concentrations of Esomeprazole and lansoprazole, since inhibitory effects were observed when increasing the dose, and an even higher toxicity was measured when prolonging the exposure to 48 h. This is in line with previous studies, which showed that the inhibition should last for at least 24 h [20]. On the other hand, long-term PPI therapy has been shown, in preclinical and clinical studies, to induce side effects, including neuroendocrine tumors and hyperplastic gland polyps [60]. Another study has shown larger risks (+39%) of prostate cancer mortality in association with PPIs [61]. Consequently, PPI treatment should be carefully considered as a novel anticancer strategy, but specific tumor types need to be selected on the basis of the real efficacy of these drugs. In line with other studies, discrepancies between in vitro and in vivo results might exist because the tumor microenvironment deeply influences the tumor metabolism and properties that are not possible to reproduce in vitro [62].

Notably, tumor pH imaging is a promising tool for investigating the role of tumor acidosis in cancer progression [38,63], immune response [38,64,65] and for assessing the treatment response in several types of cancers [40,42,65,66]. In line with the study with Dichloroacetate, the absence of extracellular tumor pH changes in the Esomeprazole-treated mice was associated with a lack of therapeutic response, hence resistance to this drug. Further studies are needed to evaluate the in vivo effect of more potent PPIs, and explore other cancer types, too.

## 5. Conclusions

This study showed that PPIs can affect in vitro cell survival and modify extracellular pH values towards less acidic values in both PC3 and LNCaP human prostate cancer cell lines. However, their efficacy is lost when treatment is performed on acidic-conditioned PC3 cells and in animal tumor models upon long-term treatment with Esomeprazole, thereby highlighting the importance of in vivo and longitudinal tumor acidosis quantification for a proper therapeutic assessment of these drugs. Therefore, MRI-CEST tumor pH imaging with Iopamidol appears to be a robust, non-invasive imaging approach for assessing the in vivo treatment response to novel cancer therapies that target tumor acidosis and metabolism.

## Data Availability

The data presented in this study are available from the corresponding author on reasonable request.

## Supplementary Materials

The following supporting information can be downloaded at: https://www.mdpi.com/article/10.3390/cancers14194916/s1, Table S1: List of primers for RT-PCR quantification of V-ATPase; Figure S1: MTT results for LNCaP human prostate cancer cell line in normoxia and hypoxia; Figure S2: pH results for LNCaP human prostate cancer cell line treated in normoxic condition; Figure S3: whole blot of V-ATPase WB assays in PC3 and acidic-conditioned PC3 cells.
