# Supplementary material for: In Vivo MRI-CEST Tumor pH Imaging Detects Resistance to Proton Pump Inhibitors in Human Prostate Cancer Murine Models
Source: Cancers (Basel). 2022 Oct 7;14(19):4916. doi: 10.3390/cancers14194916 (PMC9563279; doi:10.3390/cancers14194916)
Supplement: Supplementary file 1 [file cancers-14-04916-s001.zip › cancers-1922259-supplementary.pdf]

SUPPLEMENTARY MATERIAL

Table S1. List of primers for RT-PCR quantification of V-ATPase

Gene		*sequence*
**ATP6V1A**	Fw	GCCCATTCTACAAGACAGTAGG
	Rev	CTCCCATGTGCTCACGAATAA
**ATP6V0A3**	Fw	ATGACCTTCCTCATCTCCTACT
	Rev	GCTGCAGAAACGGGAAGA
**GAPDH**	Fw	AGCCACATCGCTCAGACAC
	Rev	CTCCATGGTGGTGAAGACG

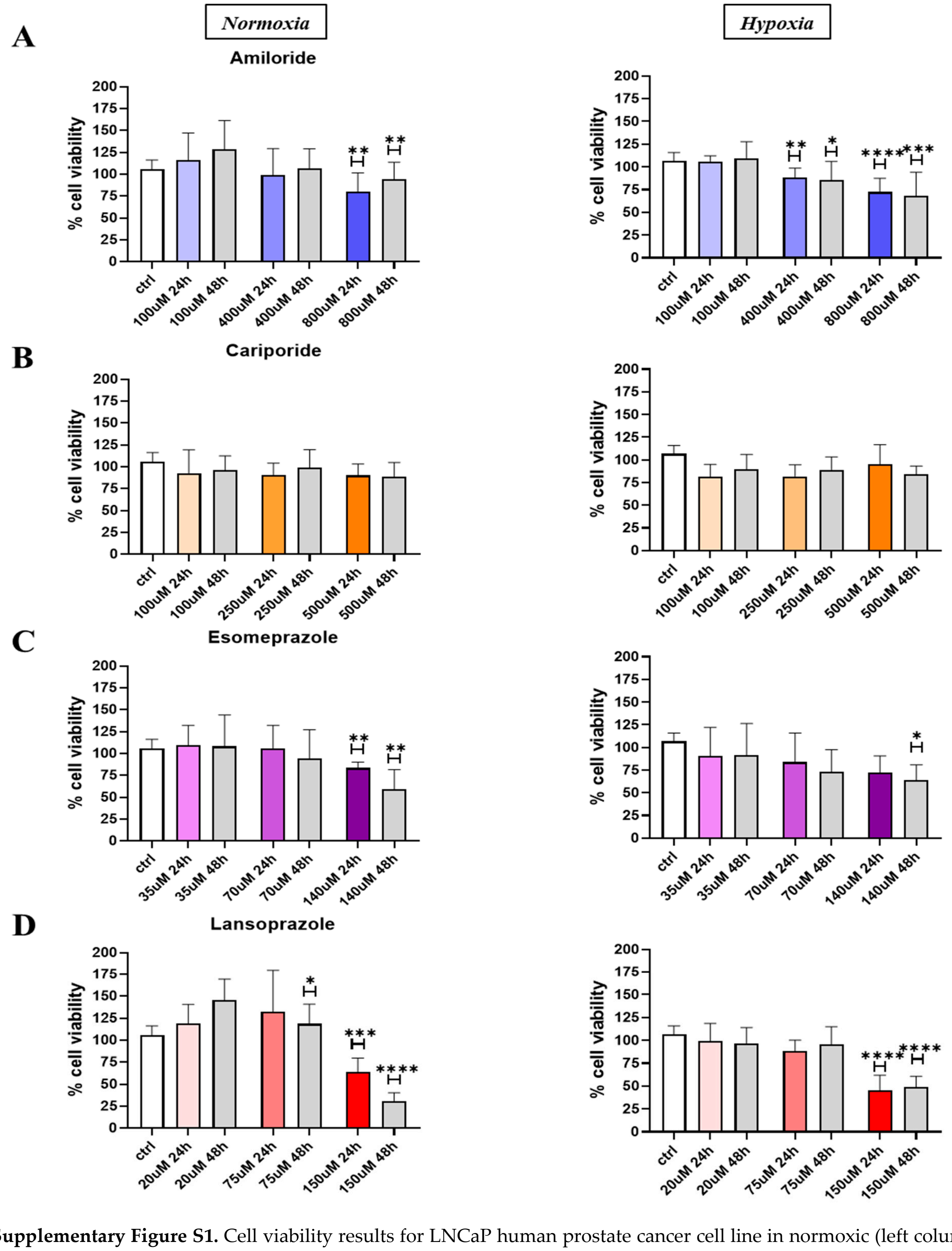

Supplementary Figure S1. Cell viability results for LNCaP human prostate cancer cell line in normoxic (left column) and hypoxic (right column) conditions at 24 or 48 hours after treatment with Amiloride (**A**), Cariporide (**B**), Esomeprazole (**C**) and Lansoprazole (**D**), respectively, at several concentrations. All drug concentrations are expressed in µM with the colour intensity matching the increasing concentration for the 24h treatment condition. The statistic differences resulted from ANOVA analysis with Bonferroni post-hoc correction against the control group tested at the two different time points * *p value* < 0.05; ** *p value* < 0.01; *** *p value* < 0.001; **** *p value* < 0.0001).

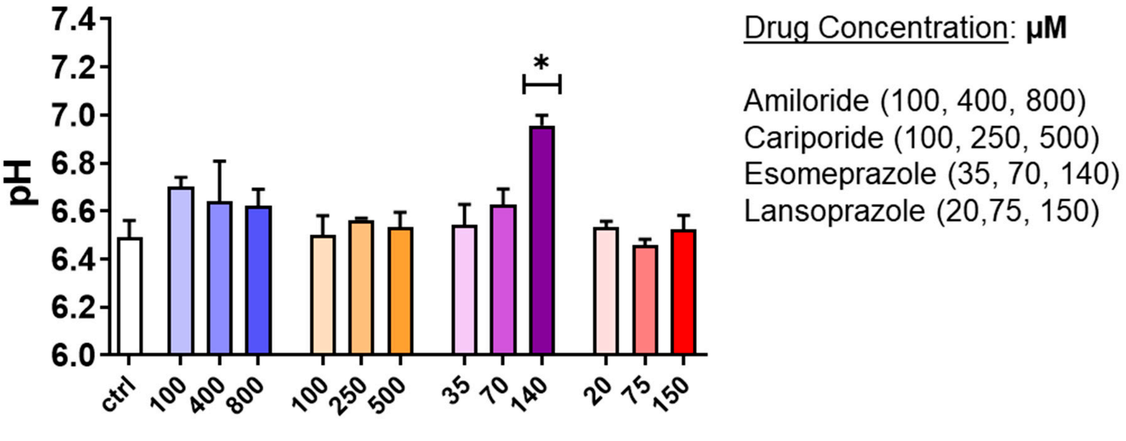

Supplementary Figure S2. Extracellular pH measurements for LNCaP prostate cancer cells after 24h treatment with Amiloride, Cariporide, Esomeprazole and Lansoprazole at several concentrations in normoxia condition. The statistic differences resulted from ANOVA with Bonferroni post-hoc correction (* *p value* < 0.05).

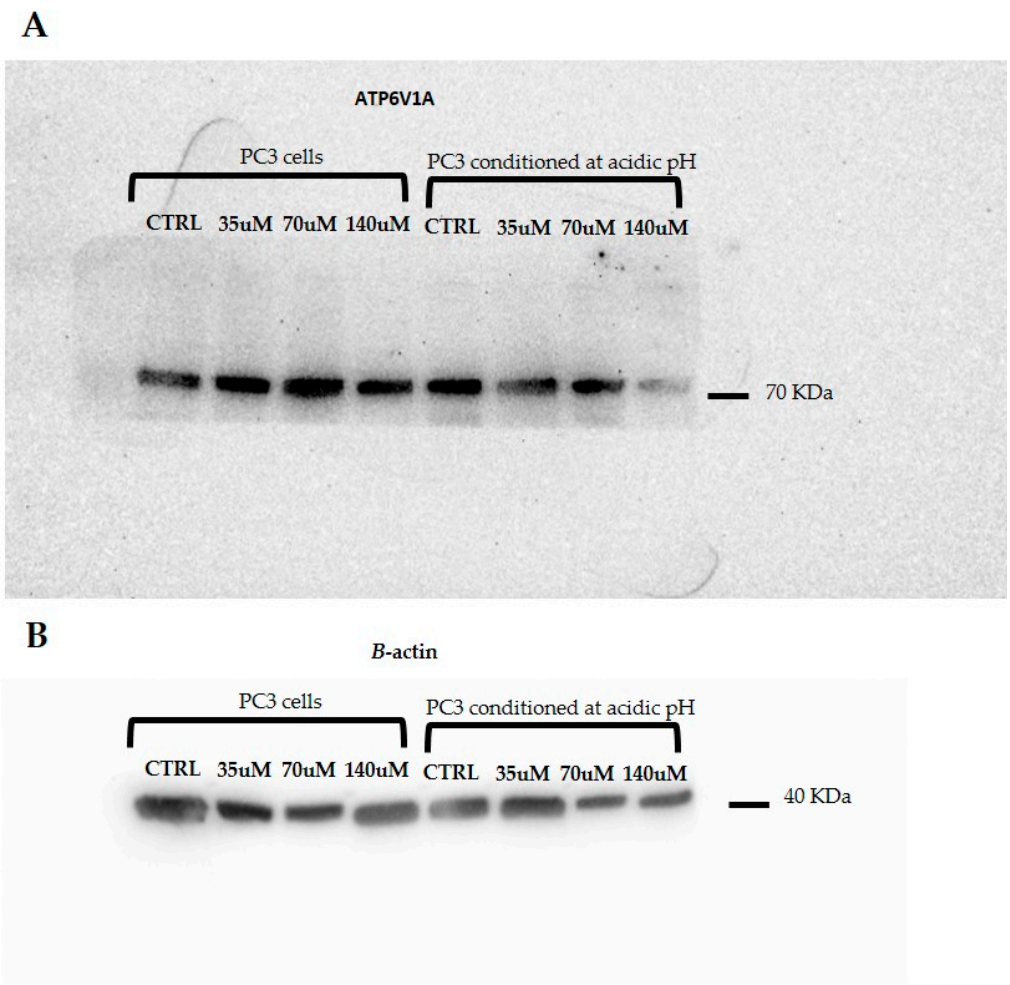

Supplementary Figure S3. Original WB pictures for V-ATPase (**A**) and Actin proteins (**B**) of normal PC3 cells and PC3 conditioned at acidic pH after 24h Esomeprazole treatment (35-70-140μM).
